# Rhino-orbito-cerebral mucormycosis in patients with uncontrolled diabetes: A case series

**DOI:** 10.1016/j.ijscr.2020.07.011

**Published:** 2020-07-15

**Authors:** Fatimah Al Hassan, Marwah Aljahli, Fadel Molani, Ali Almomen

**Affiliations:** a Department of Surgery, King Fahad Specialist Hospital, Al Muraikebat Area, Ammar Bin Thabet Street, PO Box 15215, Dammam, 31444, Saudi Arabia; b Department of Medical Imaging, King Fahad Specialist Hospital, Al Muraikebat Area, Ammar Bin Thabet Street, PO Box 15215, Dammam, 31444, Saudi Arabia

**Keywords:** Mucormycosis, Diabetes mellitus, Paranasal sinuses, Mucorales, Zygomycosis

## Abstract

**Introduction:**

Mucormycosis is a rare, aggressive, and invasive disease with a fatal outcome. It most commonly affects patients with compromised immunity, particularly those with poorly controlled diabetes. We present a case series of patients with uncontrolled diabetes and mucormycosis.

**Presentation of case:**

We present a series of three patients with uncontrolled diabetes, with main symptoms of paranasal sinusitis, nasal discharge, ophthalmic changes, and facial nerve involvement. Diagnoses of mucormycosis were made via microbiological testing and computed tomography. These cases were managed by combination therapy of tight glycemic control, urgent endoscopic sinus debridement, and antifungal therapy.

**Discussion:**

Diagnosing rhino-orbito-cerebral mucormycosis requires a high degree of suspicion and both microbiologic and microscopic evidence. Better clinical outcomes can be obtained by combining medical and surgical management.

**Conclusion:**

We describe our experience in handling three cases of poorly controlled diabetes with rhino-orbito-cerebral mucormycosis.

## Highlights

• Mucormycosis is a rare disease and is often fatal in the immunocompromised.
• We present a series of 3 patients with poorly controlled diabetes and mucormycosis.
• Diagnosing mucormycosis requires microbiologic and microscopic evidence.
• Combined medical and surgical management yields better outcomes for mucormycosis.

## 1 Introduction

Mucormycosis is a potentially fatal opportunistic infection caused by saprophytic fungi (Phycomycota, Zygomycota) of the Mucorales order and the Mucoraceae family, found in residues of plants, soil, and decaying vegetation. The fungi become pathogenic when individuals with compromised cellular or humoral immunity inhale fungal spores through the nose, mouth, or lacerations of the mucosa in the oral or nasal cavity. Those with diabetes mellitus are at highest risk [1]. Mucormycosis can manifest in many different clinical forms, including a rhinocerebral form, in the pulmonary system, central nervous system, gastrointestinal system, and other parts of the body. Rhinocerebral mucormycosis is subdivided into 3 groups: rhinomaxillary, rhino-orbital, and rhino-orbito-cerebral mucormycosis [2]. Symptoms of rhinocerebral mucormycosis are rhinorrhea, headache, intranasal or intraoral black necrotic areas, and epistaxis. Extensive forms of the disease include ophthalmia and cranial nerve involvement [1,2]. A detailed history and examination combined with histopathology can confirm the diagnosis. Aggressive management is essential to prevent lethal outcomes, and its prognosis is directly related to early detection and initiation of treatment [3].

## 2 Method

A case series, of 3 patients known to have uncontrolled diabetes mellitus diagnosed with mucomycosis managed at our hospital king fahad specialist hospital, which is a tertiary hospital and referral center, covering the eastern province of Saudi Arabia of 4 million population. All patient condtion were optomise prior to surgery with systemic, broad spectrum antibiotics, antifungal and insulin. The surgeries was performed by the senior author Dr. Ali Almomen, who is consultant in rhinology and skull base surgery, with past experience more than 15 years in this subspecialty field.

Registration: researchregistry5747

This work has been reported in line with the PROCESS [4] criteria.

## 3 Case 1

A 37-year-old male known to have hypertension, dyslipidemia, and newly diagnosed diabetes mellitus on medication was referred from a local hospital to our hospital as a case of maxillary and ethmoidal sinusitis with orbital cellulitis suspected of mucormycosis. Five days prior to his presentation, he complained of upper respiratory tract symptoms followed by greenish to blackish secretions from his right eye and discoloration of the skin of the right eyelid and cheek, associated with facial pain and swelling of the corresponding cheek. While hospitalized, he started to have a fever without chills and dysphagia mainly to solid food for four days. There was no history of head trauma, limb weakness, vomiting, or loss of consciousness. He had a history of good compliance to his diabetic treatments.

Physical examination on the day of admission showed him to be afebrile, with stable vital signs, and conscious but drowsy; for that, elective intubation was performed. He had right eyelid edema, partial eyelid necrosis, proptosis, blackish discoloration of the right side of the face (Image A), necrotic hard and soft palate, and a necrotic left hypopharyngeal wall. An ophthalmologic evaluation revealed a right fixed dilated pupil with a right afferent pupillary defect, no light perception, early retinal hemorrhage, total retinal detachment, and retinal necrosis. He was transferred to the intensive care unit (ICU) for observation. The patient underwent an urgent paranasal computed tomography (CT) scan without contrast which showed bilateral maxillary antramucosal thickening. He was started empirically on 10 mg/kg/day amphotericin B (liposomal) and 200 mg posaconazole orally every 6 h, in addition to ceftriaxone and clindamycin after septic workup. He was also started on an insulin infusion.

Functional endoscopic sinus surgery was performed as an emergency intervention. Intraoperative findings showed necrotic mucosa of the right maxillary sinus posterior wall, necrotic mucosa, and bony defects of the right sphenoid sinus walls. His tissue culture was positive for zygomycetes (*Absidia corymbifera*). The patient showed a decreased level of consciousness; as such, he underwent brain CT and magnetic resonance imaging (MRI) which showed acute infarction of the right anterior temporal lobe. The cerebral convexity level showed watershed infarction between the right middle and posterior cerebral arteries, progression of the disease with extension into the right cavernous sinus, and involvement of the right internal carotid artery wall. It also showed a ruptured right eye globe. The patient’s condition further deteriorated, and he passed away.

## 4 Case 2

A 47-year-old female with poorly controlled diabetes mellitus was referred to our hospital from a local hospital as a case of diabetic ketoacidosis with suspicion of mucormycosis. She presented with right-sided facial swelling and pain with loss of vision in the right eye. Her symptoms started three weeks before presentation.

Upon physical examination, the patient was conscious, alert, and oriented with a normal gait. There was black necrotic debris in the nasal cavity and multiple ulcers in the hard palate. An ophthalmologic examination showed complete loss of vision in the right eye with paralysis of all extraocular muscles and a fixed dilated pupil. Other significant features included decreased sensation of the right side of the face, absence of wrinkles of the right half of the forehead, drooping of the right angle of the mouth, and drooling. According to House-Brackmann grading, her condition was graded as unilateral facial nerve VI palsy. Cranial nerve examination revealed involvement of cranial nerves II, III, IV, VI.

**Fig. 1:**
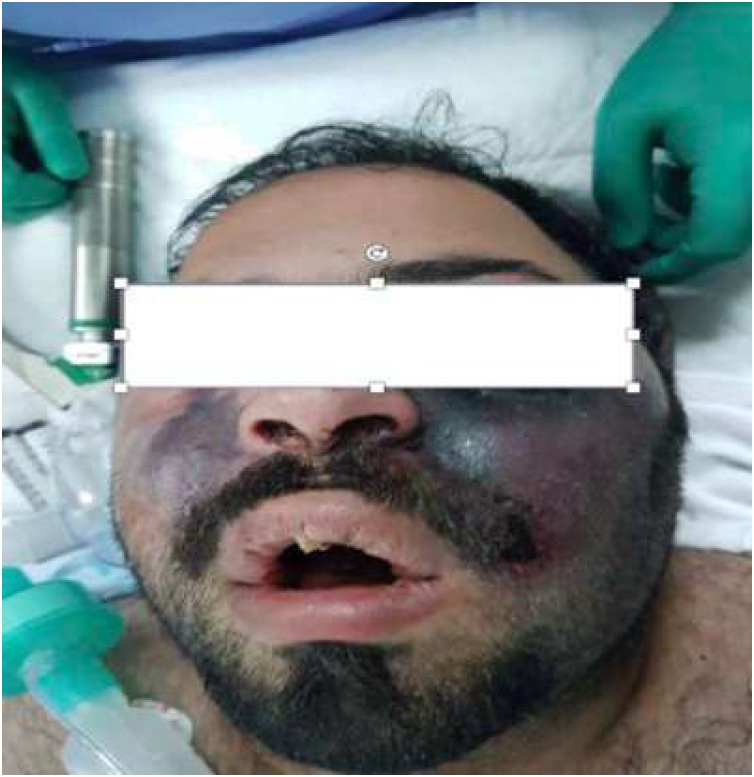
Physical sign of patient case 1.

CT without contrast showed moderate mucosal thickening of the right maxillary antrum with extension into the right nasal cavity. The antrum showed complete opacification with hyperdense contents (Fig. 1A) and a CT venogram at the level of the cavernous sinus (Fig. 1B) showed no enhancement of the right cavernous sinus (arrow), consistent with cavernous sinus thrombosis. The patient underwent urgent endoscopic sinus debridement and was managed with insulin infusion, 10 mg/kg/day amphotericin B (liposomal), and 200 mg posaconazole orally every 6 h in addition to ceftriaxone and clindamycin after septic workup. Fungal culture was positive for mucormycosis. The patient was transferred to the ICU where she developed multiple brain infarcts and cerebral artery occlusions. Unfortunately patient further deteriorated and died.

## 5 Case 3

**Fig. 2:**
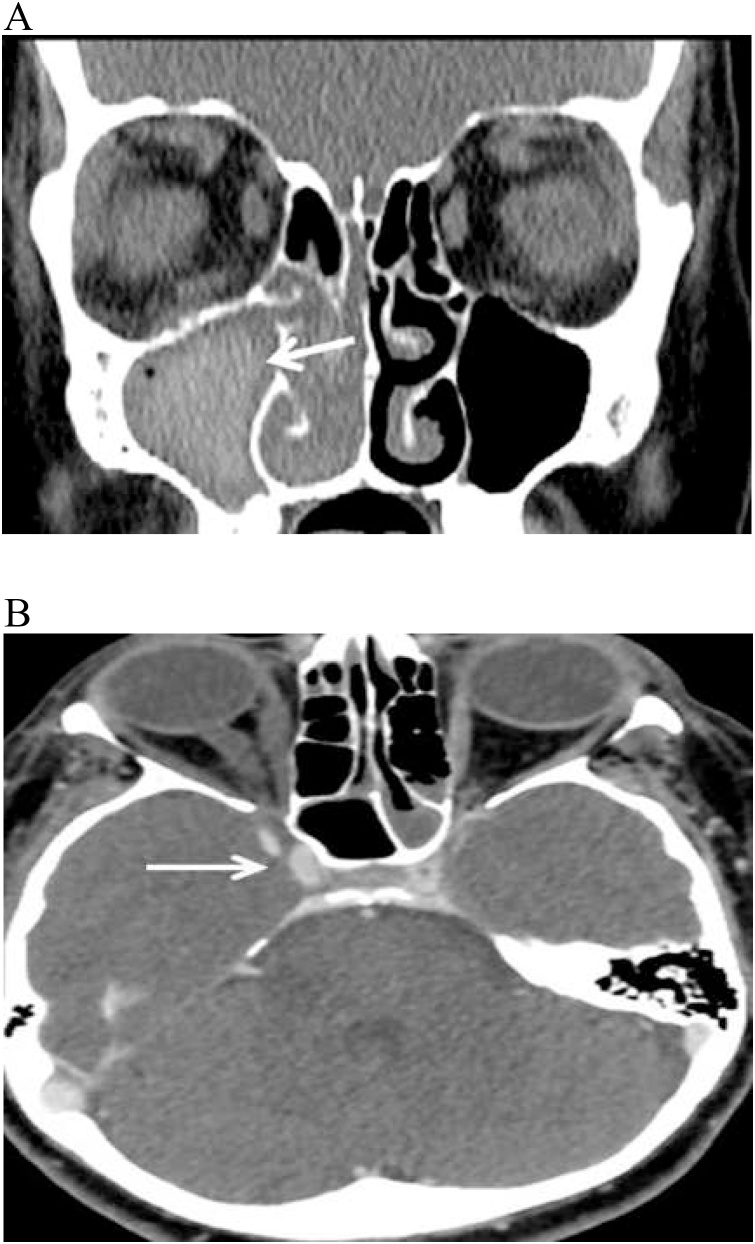
A: CT without contrast showing moderate mucosal thickening. B: CT venogram shows absence of enhancement of the right cavernous sinus.

**Fig. 3:**
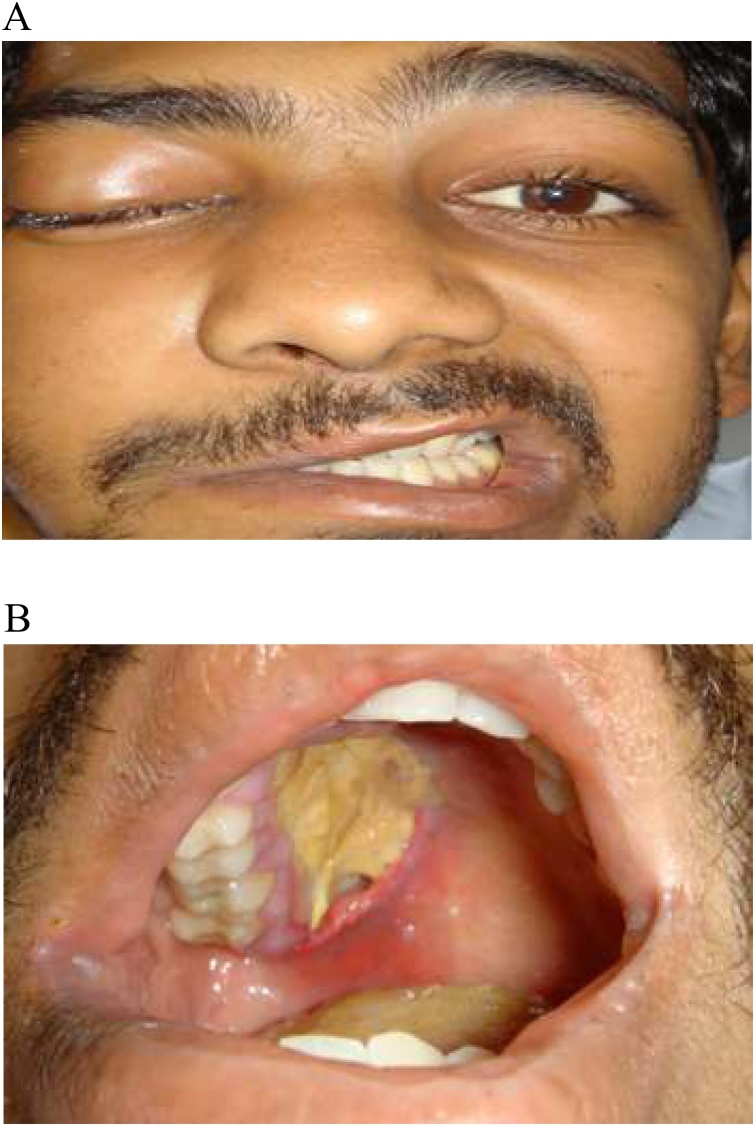
A: Facial nerve involvment in case 3. B: Big necrotic ulcer in the hard palate.

A 30-year-old male with poorly controlled diabetes was referred to our hospital presenting with headache, fever, right-sided facial pain and numbness, and an inability to open the right eye. Upon physical examination, the patient was unable to open his right eye. There was mild maxillary tenderness, a large necrotic ulcer in the hard palate (Fig. 2A), and inflamed black mucosa was noted over the middle turbinate. An ophthalmic examination showed decreased visual acuity in the right eye associated with ptosis, a fixed dilated pupil, and restricted extraocular movements. The left eye was normal. A cranial nerve evaluation revealed drooping of the angle of the mouth, drooling, absence of wrinkles in the right half of the forehead (Fig. 2B), no corneal sensation, and no light perception. A clinical suspicion of rhino-orbito-cerebral mucormycosis was established and the patient was started empirically on intravenous cefoperazone sulbactam, metronidazole, and amphotericin B (1 mg/kg), with local eye drops, insulin infusion, and close monitoring of arterial blood gases and electrolytes. CT, MRI, and an MR venogram confirmed the diagnosis of acute rhino-orbito-cerebral mucormycosis with cavernous sinus thrombosis. The patient underwent urgent endoscopic sinus debridement of the ethmoid, maxillary, and sphenoid sinuses. The patient tolerated the procedure well. Tissue culture confirmed the presence of mucormycosis. The patient was discharged after 6 weeks of intensive medical and surgical managements, patient was lost in follow-up (Fig. 3).

## 6 Discussion

Mucormycosis is defined as a range of infections caused by fungi known as zygomycetes, which reproduce sexually through zygospores. The Mucorales order of zygomycetes produces a series of aggressive clinical manifestations in different parts of the human body when immune defenses are extremely low. They most commonly affect patients with poorly controlled diabetes mellitus, especially during ketoacidosis attacks, which corresponds to 88% of reported cases of rhinocerebral mucormycosis. Other immunocompromised patients at risk are those with malignancies, transplanted organs, or long-term immunosuppressive or corticosteroid treatment [5].

If a healthy immunocompetent individual inhales these fungal spores through the nasal passage or oral cavity, they will not cause immediate or latent harm as the phagocytic response will limit its spread. The opposite process happens in patients with low polymorphonuclear leukocytes, enabling these fungal spores to germinate, develop hyphae, and locally infect the paranasal sinuses. The disease can progress and spread to surrounding structures: inferiorly to the palate, laterally into the cavernous sinus and the orbits, and cranially into the brain. It can invade the arterial lamina and give rise to thromboembolisms and infarctions of involved tissues. The consequences of this fungal spread can include orbital cellulitis, orbital apex syndrome, cerebritis or brain abscess, and death [6].

Diabetes is the most commonly known risk factor for mucormycosis, especially during ketoacidosis. Ketones facilitate the fungi to utilize and produce ketoreductase, which facilitates its growth. Ketoacidosis and hyperglycemia also directly contribute to the risk of mucormycosis by 4 mechanisms: 1) distribution of iron sequestration due to hyperglycation of iron which alters the host defense system, 2) enabling tissue penetration by expressing the cell receptor GRP78 which binds to Mucorales species through the direct effect of hyperglycemia and indirectly by increasing free iron levels, 3) impairing phagocytic functions and reducing the efficiency of chemotaxis, and 4) enhancing fungal survival through iron dissociation from sequestering proteins [2].

Clinically, mucormycosis is characterized by rhinitis with granular and purulent discharge, nasal ulceration, black spots of infarcted mucosa, paranasal sinusitis, epistaxis, facial pain with swelling, headache, ophthalmoplegia with blindness, proptosis and orbital cellulites, hemiplegia or stroke, and decreased mental function [3,7].

A diagnosis of rhino-orbito-cerebral mucormycosis requires a high level of suspicion, positive microbiological cultures, and microscopic evidence. CT scans of patients with rhino-orbito-cerebral mucormycosis can show simple sinusitis, but a negative CT scan does not necessarily rule out mucormycosis. MRI is more sensitive than CT in detecting orbital and central nervous system involvements [8].

A treatment strategy should start with elimination of predisposing factors and stabilization of the patient’s condition, as in our series, the 3 patients were managed in intensive care unit under the supervision of senior intensivist, they received systemic, broad spectrum anti-bacterial and antifungal to control suspected infection, insulin to optimize blood sugar, and other system review as needed. Excising necrotic tissue help in eliminating invasive fungi that systemic antifungals cannot reach, but the degree of debridement during surgery depends on the surgeon’s decision and frozen section findings of the debrided necrotic tissue [7]. Antifungal therapy with amphotericin B is the standard therapy for mucormycosis at a dose of 1–1.5 mg\kg\day. Based on clinical response, it can be used for several weeks with caution to nephrotoxicity. However, lipid formulations of amphotericin B can be used for longer

periods of time and in higher doses as it has fewer side effects. Posaconazole is an alternate drug of choice if the patient is resistant to amphotericin B, or it can be used as a combination therapy with liposomal amphotericin B. Amphotericin B lipid complexes act primarily as a cytochrome P-450 3A4 [9,11]. The combination of medical and surgical management increases the rate of survival from 57.5%–78% compared to only medical treatments [10].

## 7 Conclusion

Rhino-orbito-cerebral mucormycosis is a rapidly progressive fatal infection in patients with poorly controlled diabetes. A positive microbiological test confirms the diagnosis, but one needs a strong sense of suspicion to first detect the disease. Combination therapy including controlling blood sugar, urgent endoscopic sinus debridement, and antifungal treatment is mandatory to minimize the fatal outcome of this invasive and aggressive disease. We recommend to all clinicians who deals with similar patient to have a high index of suspicion and early intervention for better outcome and to minimize the morbidity and mortality of such cases, our paper is platform for future recaches to study the outcome and prognosis of Mucormycosis.

## Declaration of Competing Interest

All authors have declared that no financial support was received from any organization for the submitted work. All authors have declared that they have no financial relationships at present or within the previous three years with any organizations that might have an interest in the submitted work. All authors have declared that there are no other relationships or activities that could appear to have influenced the submitted work. All authors have declared that no financial support was received from any organization for the submitted work.

## Sources of funding

No financial support was obtained.

## Ethical approval

Case series have no ethical approval in our institution.

## Consent

Consent was obtained by all participants in this study.

## Author contribution

Fatimah Alhassan: data collection, manuscript draft, final edit.

Marwah Aljahli: data collection.

Fadehl Molani: supervision, critical version of radiological finding.

Ali Almoumen: Operating surgeon, supervision, critical revision of the manuscript

## Registration of research studies

1. Name of the registry: IJS Publishing Group Ltd.
2. Unique identifying number or registration ID: researchregistry5747.
3. Hyperlink to your specific registration (must be publicly accessible and will be checked): https://www.researchregistry.com/browse-the-registry#home/registrationdetails/5ef370b1189ff20017d1cdde/.

## Guarantor

Ali Almoumen.

## Data availability

The data used to support the findings of this study are included within the article, and they are available from the corresponding author upon request.

## Methods

This work has been reported in line with the SCARE and PROCESS criteria.

## Provenance and peer review

Not commissioned, externally peer-reviewed.
